# Challenges and Opportunities for Equity in US School Meal Programs: A Scoping Review of Qualitative Literature Regarding the COVID-19 Emergency

**DOI:** 10.3390/nu15173738

**Published:** 2023-08-26

**Authors:** Kaitlyn Harper, Bree Bode, Kritika Gupta, Ally Terhaar, Aysegul Baltaci, Yuka Asada, Hannah Lane

**Affiliations:** 1 Department of Environmental Health and Engineering, Bloomberg School of Public Health, Johns Hopkins University, Baltimore, MD 21218, USA; 2 Michigan Fitness Foundation, Lansing, MI 48906, USA; 3 Department of Nutrition & Hospitality Management, University of Mississippi, Oxford, MS 38677, USA; 4 Department of Behavioral Science and Health Education, College for Public Health and Social Justice, Saint Louis University, St. Louis, MO 63103, USA; 5 Division of Epidemiology and Community Health, School of Public Health, University of Minnesota, Minneapolis, MN 55455, USA; 6 Community Health Sciences, School of Public Health, University of Illinois Chicago, Chicago, IL 60607, USA; 7 Department of Population Health Sciences, School of Medicine, Duke University, Durham, NC 27708, USA; hannah.lane@duke.edu

**Keywords:** food security, school meals programs, summer food service program, COVID-19

## Abstract

The emergency school meals program provided free meals to children in the United States (US) during COVID-19-related school closures. This scoping review aims to synthesize the existing qualitative empirical evidence published between March 2020 and January 2023 on the operations and utilization of emergency school meal programs during the pandemic. Qualitative, US-based peer-reviewed literature was collected from three sources: (1) parent review of all federal nutrition assistance programs; (2) systematic search of four databases; and (3) manual search of grey literature. Identified scientific articles and grey literature reports (*n* = 183) were uploaded into Covidence and screened for duplicates and inclusion/exclusion criteria. Our final sample included 21 articles/reports, including 14 mixed methods and seven qualitative-only projects. Articles spanned all U.S. states. Articles had similar research questions to understand school meals and/or general food access during COVID-19, with an emphasis on long-term policy implications. Hybrid deductive/inductive analytic coding was used to analyze data, utilizing domains from the Getting to Equity Framework (GTE). GTE considers multi-level factors that influence nutrition behavior while centering more equitable pathways to improve nutrition security and reduce adverse health. Findings were sorted into two categories: operational challenges during the pandemic and solutions to address inequities in school meal distribution during and after the pandemic, particularly during school closures such as summers or future emergencies. Key challenges related to supply chain issues, safety, and balancing families’ needs with limited staff capacity. Programs addressed equity by (a) reducing deterrents through federally issued waivers and increased communications which allowed the serving of meals by programs to families who previously did not have access, (b) building community capacity through collaborations and partnerships which allowed for increased distribution, and (c) preparing and distributing healthy options unless barriers in supply chain superseded the effort. This review highlights the importance of emergency school meal programs and provides insights into addressing challenges and promoting equity in future out-of-school times. These insights could be applied to policy and practice change to optimize program budgets, increase reach equitably, and improve access to nutritious meals among populations at highest risk for nutrition insecurity.

## 1. Introduction

Children living in low-income households, those living in rural areas, and those identifying with racial and/or ethnic minority groups are among the most likely groups to experience inequitable access to nutritious food and associated adverse health outcomes such as food and nutrition insecurity. Food and nutrition insecurity (i.e., limited or uncertain availability of nutritionally adequate, culturally preferred, and safe foods) [1,2,3,4,5] is inextricably linked with poverty and structural racism, the “system by which public policies, institutional practices, cultural representations, and other norms work in various, often reinforcing ways” to offer advantage to white people over Black and Brown people [6]. Food insecurity is complex and there is no one ideal solution; however, any approaches to close food access gaps should center on social and structural determinants of health, including racism and other forms of oppression and discrimination [7,8,9,10].

Federally funded school meal programs—including the US Department of Agriculture (USDA)’s National School Lunch Program (NSLP), School Breakfast Program (SBP), and Summer Food Service Program (SFSP)—can serve as health equity-focused policy solutions to advance food security by providing a source of year-round nutritious and consistent meals for all children. Roughly 30 million children and adolescents currently participate in school meal programs, including approximately 20 million low-income students who receive meals for free or at a reduced price [11]. However, the potential impact of school meal programs on food insecurity and poverty is stifled by limited participation—prior to the COVID-19 pandemic, only two thirds of eligible children participated in the NSLP, and approximately half of eligible children participated in the SBP [11,12] during the school year. In the summer months, when food insecurity increases and weight gain accelerates among children, only 14% of eligible children participated in school meal programs prior to COVID-19 [13,14]. In the past few decades, research has accumulated description of many implementation barriers (e.g., limited site access in high-need areas, stigma, limited variety or cultural acceptability, complex reimbursement, stringent regulation, and historic disinvestment in school infrastructure) that have led to this participation gap [15,16,17]. Yet, until the onset of COVID-19, there have been minimal federal efforts to address these barriers.

When COVID-19 forced broad school closures in March 2020, USDA implemented nationwide waivers that allowed the continuation by school districts of providing meals to all children while mitigating virus spread [18]. The waivers enabled flexibility in federal regulations and formal agreements so that school food service directors could make more locally driven decisions. Examples of operational changes facilitated by the waivers included serving meals to all students, regardless of income eligibility; serving meals in non-congregate settings (e.g., grab-and-go or drive-through meal service) and serving multiple meals at a time; expanding hours of meal service; and waiving certain meal pattern requirements to combat food shortages and respond to the needs of students and families. Recent research has shown that during 2020 closures, the waivers allowed the serving of approximately 8.9 times more meals (1.3 billion meals) than in the summer of 2019 [19], increasing spending from USD 475 million to USD 4.1 billion dollars [19,20,21,22].

During the period that the waivers were in effect (primarily 2020–2022), researchers across the US collected empirical implementation data, which is primarily qualitative, to examine how waiver operations worked across a variety of policy, cultural, and geographical contexts. The goal of this scoping review was (1) to describe the scope of existing qualitative literature on emergency school meal programs, including operations, utilization, and challenges to meal distribution; and (2) to evaluate the degree to which emergency school meal programs addressed challenges and optimized reach to all children using the Getting to Equity (GTE) framework. GTE, first published in 2018 [7] and applied specifically to food insecurity and school meals in 2020 [8], provides solution-oriented guidance to inform such approaches, and to eliminate structural factors that inhibit food and nutrition security for all, especially low-income children and families.

## 2. Materials and Methods

**Figure 1:**
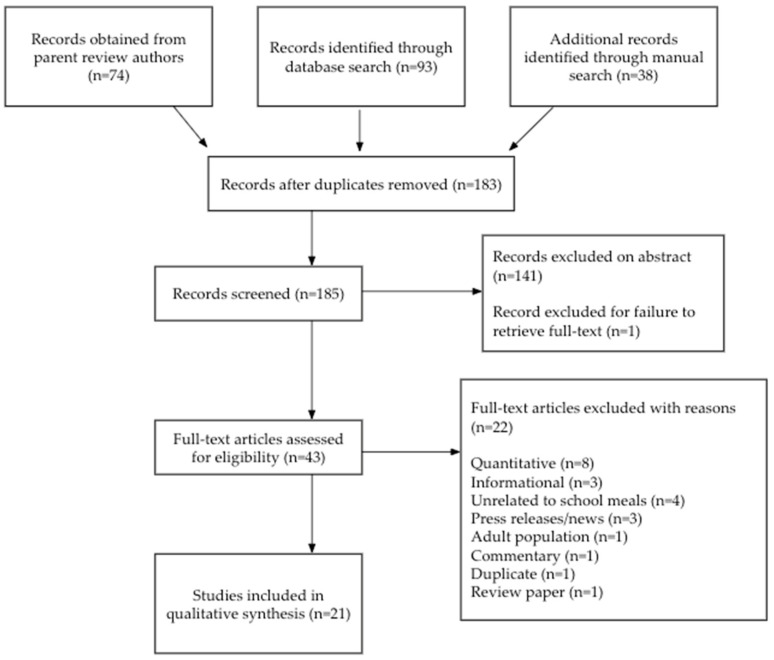
PRISMA flowchart of the study selection process.

We conducted a scoping review of qualitative literature in the United States (US) that sought to characterize the implementation of school meal program operations across the country under the COVID-19 waivers. Scoping review methods were selected given the limited understanding of the evidence [23]. We followed PRISMA Guidelines to ensure that our process was rigorous and systematic [24] (Figure 1) and conducted a thematic analysis of included studies.

### 2.1. Search Strategy and Study Selection

We included peer-reviewed articles and grey literature in this review. For peer-reviewed articles, we leveraged the search strategy of a separate scoping review [25] (referred to hereafter as the “parent” review) which sought to characterize the research on all federal nutrition assistance programs (e.g., Supplemental Nutrition Assistance Program (SNAP); Special Supplemental Program for Women, Infants, and Children (WIC); school meal programs such as SNLP, SBP, SFSP). The articles in the parent review were extracted from four databases—PubMed, CINHAL, Scopus, and ProQuest Health Management—between March 2020 and March 2022 (Supplementary File S1). The parent review authors provided us articles specific to School Meal Programs (*n* = 74). We replicated the search on 7 January 2023 and identified 93 additional articles.

For grey literature reports, we conducted two Google searches (25 October 2022, and 7 January 2023) using search terms most relevant to the objectives of this review and different combinations of the terms “school meals”/“waiver”/“covid”. The searches yielded sources such as newspaper articles; blogs; federal and state websites; reports; and other informational websites. We followed recommendations of our Health Sciences Librarian and reviewed the first 100 hits for both dates. For the grey literature search, 164 of the 200 hits were excluded upon title review because they were informational websites or informational links to social media webpages. In the abstract and full text review, we included US-based qualitative or mixed methods papers that described barriers/facilitators of serving school meals and excluded grey literature if they were quantitative only, commentaries, or newspapers/magazines.

We used Covidence, a web-based collaboration software platform that streamlines the production of systematic and other literature reviews [26], to extract and organize data from each article. We created a standardized extraction form, including aim, participant characteristics, characteristics of families served, study location, recruitment method, type of data collection, and type of data analysis. The standard form was also used to extract results related to program operations and implementation, including operational challenges and utilization of the USDA waivers.

Two members of the research team performed title and abstract screening of all peer-reviewed and grey literature (*n* = 184) and removed irrelevant or ineligible articles (*n* = 141). Next, three members of the research team performed full-text screening (*n* = 41). During the full text screening, we reviewed reference lists and similar article lists within the search engines to ensure an exhaustive search. From this, we identified two additional articles. We included any peer-reviewed articles and grey literature reports in English that examined or explored the operations of school meal programs during the COVID-19 pandemic and described waiver implementation, reach and utilization, challenges, social and structural barriers, best practices, and gaps in practices. We included primary research studies with qualitative-only research design as well as mixed methods studies that reported relevant qualitative findings. We excluded articles published in languages other than English, quantitative research studies, review articles, press releases/news articles, and commentaries. We excluded one paper (a textbook chapter) because we could not access the full text.

### 2.2. Analysis

For our first objective, we first created descriptive summaries of the results section of each paper according to the following prompts: (1) Describe the operations, reach, and utilization of the emergency meals programs; (2) Describe utilization and perceived impact of USDA waivers on the emergency meals program. Each paper was summarized by two team members to ensure thoroughness and consistency [27]. Then, one member of the research team inductively coded the summaries to identify patterns which were checked by three other members of the research team. Summaries were synthesized into key concepts or themes described below. Importantly, we did not attribute percentages or other quantitative metrics to themes across the studies, as studies were conducted over different time frames, with varied research questions, data sources, and reporting priorities; thus, published data do not reflect the full operational experiences described by each sample.

For our second objective, we performed a hybrid deductive/inductive thematic analysis of the included studies [28]. We broadly identified challenges in operations and program utilization described across studies. Then, we utilized the Getting to Equity (GTE) framework to characterize the extent to which emergency school meal programs described equity considerations in addressing these challenges [7,8]. The GTE framework was selected for this analysis because it is designed to aid practitioners and public health researchers in identifying ways to increase the equity impact when undertaking policy, system, and environmental interventions, such as school meal programs, to reduce health inequities. The GTE framework identifies four key domains that interventions should address to focus on equity: (1) increasing healthy options, (2) reducing deterrents, (3) improving social and economic resources, and (4) building on community capacity. Additionally, our review builds upon foundational definitions for these four domains applied to school meal programs by McLoughlin et al. in 2020 [8]. We wrote thematic narrative summaries for each GTE domain, then constructed themes through additional coding.

## 3. Results

Of the 21 records included in the synthesis, 16 (84%) were original peer-reviewed articles and 3 (16%) were other types of records (Table 1). Methods were either mixed methods (*n* = 14, 67%) or solely qualitative (*n* = 7, 33%). School meals were the primary focus of 10 (48%) articles, while 11 (58%) integrated school meals as a secondary focus (e.g., primary focus on the role of teachers, Registered Dietician Nutritionists, and school food employees or variables such as food safety). The studies collectively spanned all US states and Washington DC and five US territories. California, Ohio, and New York were part of multiple studies. The communities served included urban alone (*n* = 7, 33%), rural alone (*n* = 1, 5%), urban, suburban, and rural together (*n* = 4, 16%), urban and rural together (*n* = 4, 21%), and locations that were not specifically named (*n* = 5, 26%). Participants of interviews and focus groups were emergency food task force members, school food service directors, school superintendents, parents, community partner organization representatives, vendors, Latino immigrants from agricultural communities, and state agency directors. Additionally, nine (43%) articles explicitly listed the race/ethnicity of communities served or included analyses in which races/ethnicities were of interest. The data collection protocols (i.e., interview questions, open-ended survey questions) were similar across studies and typically inquired about the COVID-19 pandemic, policy, school meals, and general food access. Only two studies (10%) included a Health Behavior Theory—namely the Social Ecological Model [29,30]—and five (24%) included a guiding approach or framework such as GTE [7], Community-Based Participatory Research [31,32], Consolidated Framework for Implementation Research [33] or Sendai Framework for Disaster Risk Reduction [34]. Out of the 21 studies included in this analysis, 16 (76%) studies examined or described USDA waivers. 

### 3.1. Principal Findings

**Figure 2:**
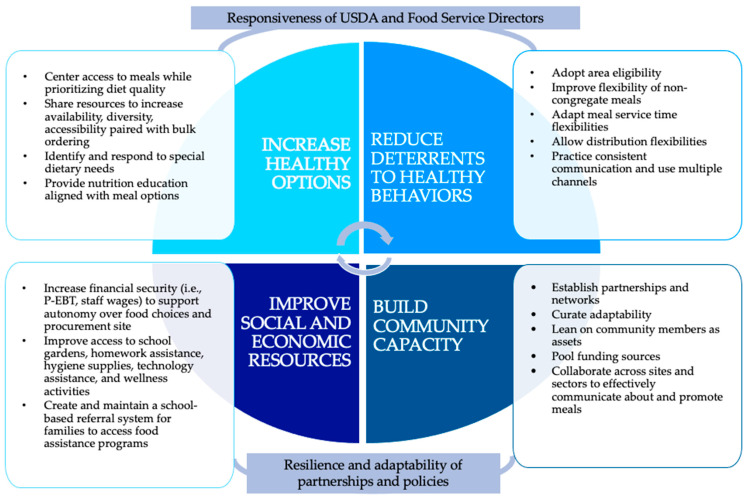
Adapted from Getting to Equity framework [7] and expanded from McLoughlin et al. [8]. Considerations are offered to mediate and/or moderate social and structural barriers to promote equitable school meal policies and program implementation during an emergency such as COVID-19.

Next, we describe themes constructed across studies that highlight challenges encountered by school meal programs during the COVID-19 pandemic; these challenges were organized using the four quadrants of the GTE framework to better understand equitable solutions: (a) Reduce deterrents, (b) Build on community capacity, (c) Improve social and economic resources and (d) Increase healthy options (Figure 2).

### 3.2. School Meal Programs Faced Both Familiar and Unfamiliar Challenges during the COVID-19 Pandemic

Almost all (81%) articles highlighted implementation challenges faced by food service directors serving emergency school meals. Some of these challenges aligned with those described in prior research on summer meal programs, but were amplified by the pandemic: limited awareness of/dissatisfaction with the program, inconvenient times for families, lack of transportation, stigma, and limited options for religious conventions or dietary needs [37,38,41,46,49,53]. Pandemic-specific challenges included supply chain issues (i.e., being unable to receive the variety of foods obtained in the past), limited communication to families about how and where to access meals, and excessive waste [37,38,39,41,45,46,47,49,50,51,52,54] Authors described how supply chain issues exacerbated family dissatisfaction—in one study, families reported stopping the use of the school meal programs because their kids refused to eat the food provided [53]. Parents in one study reported on a variety of ways that communication could have been more equitable (e.g., signage in multiple languages, more mail notifications for households without reliable Internet) [49]. A related challenge was balancing parent schedules and logistical desires with limited staff capacity. In one study, food service directors noted that they knew parents were more likely to be able to pick up meals in the evenings and over the weekend, but it was too expensive to staff in the off-hours because the union required them to pay overtime [40]. Additionally, both food service directors and families described challenges mitigating fears around disease transmission and the physical safety of both staff and families when receiving food from distribution sites [35,39,40,41,42,43,47,50,51].

Other operational challenges included staff burnout, staff illness, limited volunteer capacity, and staff feeling underappreciated [38,40,42,45,46,47,50]; difficulty predicting participation numbers, particularly as a result of competition with other nearby food distribution programs and federal programs such as Pandemic EBT (a USDA food assistance financial benefit issued to families to support buying groceries during the COVID-19 pandemic) [38,39,40,46,47,50,52]; financial deficits resulting from increased operational costs and unpredictable participation numbers [40,41,47,50]; and food safety concerns, such as maintaining the cold chain with deliveries [43]. Food service directors also described challenges related to the USDA waivers, such as lack of adequate resources or knowledge to implement the waivers and a lack of communication from federal and state agencies [39,42,48,50,51,52,54].

### 3.3. SFSP Food Service Directors Addressed Challenges with Equitable Solutions

Despite facing innumerable challenges throughout the course of the pandemic, food service directors found innovative tactics to continue meal distribution. While most studies did not explicitly describe utilizing specific tactics with the purpose of increasing equity, we conceptualize the ways in which their approaches addressed social and structural barriers and promoted equitable meal distribution using GTE domains.

#### 3.3.1. Reduce Deterrents

As described above, we used definitions of GTE domains created by McLoughlin et al. (2020) [8]. Reducing deterrents refers to removing barriers to equitable food access for people who may have been experiencing low access due to their race, ethnicity, place, role, or socioeconomic status.

The USDA waivers were the biggest tool food service directors used to address barriers to accessing free meals. For example, 15 (74%) studies described the USDA waivers as a mechanism to increase access to meals in ways that had not previously been possible. The area eligibility, non-congregate meals, meal service time flexibility, and parent/guardian pick up waivers were the most referenced [18]. These waivers enabled delivery of meals to homes or other easy to reach locations (e.g., parks, parking lots, homeless shelters, bus stops) in previously inaccessible community areas, providing meals through “grab and go” systems, and offering multiple days’ worth of food at one time at no cost to all children. Importantly, food service directors in included studies noted that the waivers enabled them to prioritize reach to high-risk subgroups, including families with transportation limitations and low-income families who lived in areas that were not normally eligible to have a site nearby or geographically difficult to access [8,39,41,50]. In one study, parents appreciated being able to access any site within a city if they wanted to, both for convenience during working hours or to reduce stigma of being seen by neighbors [50]. Across studies, food service directors had overwhelmingly positive perceptions of the waivers and expressed their continued utility in reducing deterrents post-COVID-19 pandemic.

Consistent communication was another approach used to reduce deterrents to participation. Approximately one third (36%) of studies intentionally described increased communications with families through a variety of modalities including social media, printed fliers and signs, announcements from teachers through the virtual classroom, emails from food service directors and school administrators (e.g., principals, superintendents), reminder texts, information on school and district websites, robocalls and phone banking, press releases, and hosting promotional events [8,35,39,40,42,46,49,52]. One study among parents found that these multi-modal communication strategies raised their awareness [49]. Additionally, one study described using social media to train volunteers and to share ideas and tips between districts related to implementing waivers and other state and federal policies. Although most studies noted that the main goal of communication was to reach as many families as possible, a few studies described how the programs catered their communication strategies to reach a diversity of families in their district, particularly those in Black and Brown communities. One study specifically increased communication to tailor recruitment of Black and Latino families from low-income neighborhoods to participate in meal offerings at respective sites [49]. In one study of four large urban school districts, the authors noted that two districts used advertisements with racially and ethnically diverse children and the other two districts did not [35]. Additionally, three studies described bilingual marketing for Spanish-speaking families [35,39,46]. This was important for families in one study of Latino farm workers, who noted that English-only communication was a barrier to accessing the programs [39].

Finally, although this was not a common approach, food service directors in one study described participating in stigma training as an approach to creating belonging and supporting people participating in school meals [46]. The FSD in this district also sought to reduce stigma by recruiting community members to be social media influencers, and making school meal pick up sites seem more about having family time and fun activities and incorporating a familiar community concept (e.g., promoting a farm stand) [46].

#### 3.3.2. Build on Community Capacity

Building community capacity refers to partnerships and collective efforts of school meal programs with other organizations and networks [8]. A total of 10 of 21 articles (48%) described the importance of partnerships to improve access to school meals [8,35,38,39,42,46,47,48,50,52]. Having established partnerships or networks increased the bandwidth of districts to respond to the food and nutrition security needs of the communities they served [38,52]. For example, one study noted that partners helped distribute school meals to families of first responders to address food and nutritional needs [35]. Additionally, alternative sites for meal distribution were identified to better meet the needs of community members and reduce barriers to access [38,46]. Community-based sites included homes of families who were unable to leave independently to acquire their own meals and where members of the National Guard became community assets for the meal delivery component [38].

Partnerships also expanded the reach of school meals by increasing the number of funding sources and fundraising opportunities, addressing gaps in distribution plans, and mitigating supply chain issues [35,39,42,50]. Pooling funding sources across partners created avenues for dinner meals to be served, to produce boxes to be provided alongside meal distributions, and to ensure consistency in full meal plans distributed to school children; also, they offered budget relief to buffer the effects of debt [35,39,42]. Other unique ways that partnerships expanded reach were through collaborative messaging and strategic communications about school meal programming. For example, FSDs from neighboring towns formed a partnership. Together, with the aim to reach more children with messages about meal offerings and locations, the neighboring towns cross-promoted USDA-sponsored and non-USDA-sponsored meal offerings, which was especially important for families in neighboring towns where summer meal programming was not offered. As a result, students and families who may not have met traditional eligibility requirements learned how and where to receive a meal in a town that was close to home at a time that was best for their schedules [46].

In other cases, school meals programs worked with community-based organizations and government offices to cross-promote their programs, which was “mutually beneficial” for all parties because they were able to increase participation at all partner events [39]. In one study, partnerships were not previously established across districts, and participants noted that the absence of such partnerships stunted their ability to be innovative or respond in a fully community-based manner [51]. One study also reported capacity built among multi-level decision makers within the school nutrition community through increased communication, which informed innovation of school meal dissemination. Specifically, waiver operations necessitated increased communication between USDA, state agency and local partners, and led to more discussion amongst food service directors within states or local regions which spread innovation [50]. School meal vendors noted the need for continued local coalition building and community learning as a necessary next step to build on community capacity to meet the needs of students and their families.

#### 3.3.3. Increase Healthy Options

The increasing healthy options domain refers to the mechanisms by which emergency school meal programs distributed food, how they promoted nutritious foods (e.g., through posting menus or providing recipe cards), and how they promoted overall safe access to foods [8]. Providing nutritious meals to children is the top priority for school meal programs; however, food shortages and supply chain issues sometimes hindered food service directors from accessing and/or serving these foods. Regardless, food service directors consistently looked for ways to improve the availability of healthy options. In one study, food service directors noted that, although the USDA meal pattern waiver allowed them the serving of foods that did not meet the dietary requirements, they used the waiver only when food availability was an issue in order to maintain the nutrition-related integrity of program standards [51]. In another, a food service director from a small rural district partnered with a neighboring district to purchase a walk-in refrigerator and use the process of bulk ordering to reduce costs of stocking healthier options [50]. Food service directors also worked hard to provide healthy meals particularly for children with specific dietary needs or preferences. Two studies highlighted food service director communication directly with families to accommodate student needs amid supply chain shortages and bulk transportation [46,52]. Another food service director noted that they would hand out bulk items rather than premade meals with recipe cards so that families could prepare the meals in ways that would best suit their needs [48].

#### 3.3.4. Improve Social and Economic Resources

Improving social and economic resources beyond meeting immediate food and nutrition needs is an important domain of the GTE framework. This domain refers to the ways in which school meals programs provide resources to families to help offset other costs, which in turn mitigates the effects of economic and food insecurity. In a study including parents, many participants felt that the meals independently and in combination with P-EBT increased their sense of financial security by reducing their grocery budget [50]. Food service directors offered a number of non-food resources to promote health through additional social resources, such as access to school gardens, homework assistance, hygiene supplies, technology assistance, wellness activities, and community events, such as “grill outs” [8,46,48,49]. McLoughlin et al. (2020) described ways school meal programs can improve social and economic resources through assisting with child and family needs, such as helping families access food assistance programs or access to federal stimulus funds [8]. There were fewer examples of this construct in the studies included in this review. However, three studies highlighted the importance of the waivers in improving social and economic resources primarily for Latino families. In one study of Latino immigrants living in a rural district, parents noted that school meals and Pandemic Electronic Benefit Transfer (P-EBT) were the only federal assistance programs they were able to access during the pandemic [53]. In another study, parents reported using P-EBT to accommodate picky eaters which offered parents flexibility to provide food options beyond school meals [49]. Parents from the same study also felt that P-EBT increased their autonomy over food options. In the third study of school districts that predominantly served children of Latino farm workers, food service directors reported that the waivers allowed them the accommodation of schedules of parents who often worked early in the morning until mid-afternoon; children in these families were not able to attend the summer meal program in previous years because breakfast and lunch were served during times when working parents could not bring their children to the sites [39].

Another important way that school meal programs can provide social and economic resources is through upstream solutions that work toward removing challenges for school meal staff [8,35,38,46,51,53] typically related to a need for improved wages, acknowledgment or belonging, and other benefits. Various ways that staff were supported were described in studies, including increased wages, incentives such as childcare, verbal appreciation and acknowledgement from school administrators, staff, and families, and opportunities for professional development [42,50].

## 4. Discussion

In this scoping review, we described the landscape of qualitative literature on emergency school meal programs and identified major implementation challenges impacting participation in programs [55]. Additionally, we applied the GTE framework to describe the extent to which studies reported on how challenges were addressed with considerations for health equity. The major challenges reportedly faced by school food service professionals during the COVID-19 pandemic were the pace of policy change, the navigation of ways to meet the needs of everyone involved in the changes (e.g., school staff, parents, students, vendors, etc.), as well as the adaption to fluctuations in participation throughout the course of the pandemic. School food service professionals addressed challenges using a range of actions including adopting, applying, and implementing waiver flexibilities; increasing communication through multiple avenues to increase awareness of the program; and collaborating with nonprofits, businesses, and government agencies to improve access to meals.

The GTE framework proved to be a useful tool for evaluating how emergency school meal programs utilized equitable approaches to adapt to challenges. We found that data were most robust in the *reducing deterrents* domain. Conversely, the *improving social and economic resources* domain had the least supporting data. Although the school meal programs inherently improve social and economic resources by allowing families reallocation of funds toward non-food needs (e.g., housing, bills, healthcare), the studies published during the pandemic focused mainly on reporting food service director perceptions of barriers and facilitators to serve meals (i.e., how to reduce deterrents). Additional studies focused on family perceptions of COVID-19 relief programs, including school meal flexibilities and waivers, which may have revealed more about social and economic resources. Other pre-pandemic studies have highlighted these benefits [56,57,58].

Overall, the authors of only one article in this review explicitly utilized a health equity framework as part of their methodology [8]. The authors found that while strategies to increase healthy options were prevalent, there was less emphasis on improving social and economic resources, thus potentially perpetuating inequities. The dearth of findings that specifically address equity in this body of research likely occurred because researchers were designing and conducting studies rapidly, school food services were undergoing rapid changes, and the stay-at-home order limited partnership-building or in-person communication during these studies. Thus, equity may not have been intentionally used to formulate research or evaluation questions or conduct analyses. The authors of the included studies may not have had prior experience conducting health equity-centered implementation studies [59], which have been given increased attention since the murder of George Floyd and the subsequent racial equity movement that expanded in 2020 [60,61,62]. As studies continue to be designed and conducted in relation to the implementation of school meal programs and other USDA federal nutrition assistance programs, greater focus should be given to evaluating equity and including health behavior theory to understand opportunities for policy improvement, practice-based improvements, sustainability of approaches to improve equity, and cultural acceptance of policy, systems, and environmental changes as they are related to and situated within health equity frameworks [7,63,64]. Without such a focus, nutrition policy researchers run the risk of making policy recommendations that further exacerbate, rather than solve, existing food and nutrition insecurity inequities.

Our synthesis of the literature on school meal program operations under the waivers enables recommendations to improve meal distribution during out-of-school times, particularly in the summer. Specifically, the waivers allowed programs a quick pivot and adaptation in creative ways to provide more meals for families who previously faced barriers to access. Looking forward, it is important to continue to build on local efforts to provide equitable access. This includes considering the structural barriers that hinder programs from providing access to the most vulnerable populations. Racism and other forms of systemic discrimination are built into the structure of our society [65]. It has created unequal opportunities for wealth-building over generations through systematic policies and practices such as housing and supermarket redlining, discriminatory loan-lending, unequal policing and police violence particularly targeting Black men, unequal access to healthcare and medical treatment, and unequal employment opportunities and wages for Black and Brown workers [62,65]. We recognize that it is not the role of school meal programs to address all these structural barriers, but we note that there are ways to enable better access for families who, because of systemic discrimination, may rely on school meals for food security but also have limited means to access them during out-of-school periods.

### 4.1. Implications for Programs, Policy, and Future Directions

To start, federal and state policy changes to expand area eligibility (enabling more strategic meal site placement) and maintain non-congregate meals (enabling parent pick-up and meal timing flexibilities to meet local needs) are needed. Policy changes that expand eligibility and maintain non-congregate meals seem especially realistic since—despite the challenges—emergency school meal programs through waivers and flexibilities demonstrated a tremendous value on the investment by making a pathway for increased food security as evidenced in this scoping review.

This review suggests that the USDA waivers played a significant role in increasing meal access and serving children who were previously unable to access out-of-school meals, and could improve participation during future school closures and summers. For example, the SFSP’s reach is limited by area eligibility requirements, which currently restrict the program to areas where at least 50% of children are eligible for free or reduced price school meals [66]. Relaxing these requirements could enhance the impact of the SFSP and prevent summer meal disruptions among school children. Additionally, in 2023, the Consolidated Appropriations Act established a permanent non-congregate meal service option for rural areas with no congregate meal service available [67,68]. This will remove many barriers for participation for those living in rural areas; however, barriers still exist for children living in suburban and urban areas, and expanding the Act to include such areas would provide more flexibility for parents and may improve SFSP participation in the coming years. Additionally, extending the policy to allow parent pickup of meals may increase participation due to increased convenience for parents who, for example, could pick up meals for their children on their way home from work.

Additionally, we found that supply chain issues caused a major disruption to the continuation of school meal programs. Increased support for Farm to Summer programs may help mitigate supply chain issues by creating/improving communication between food sources (i.e., farmers) and summer meal providers, as well as simplifying the process of receiving food by simplifying the process and shortening the travel distance between food source and distribution sites. Research has also shown numerous positive effects of serving local foods and providing food education through child nutrition programs, including increased participation and engagement in meal programs, consumption of healthier options, and support of local economies [69,70].

Finally, there is a need to strengthen technical assistance from state and federal agencies to school food service directors during periods of school closure such as summer to support clear, timely, tailored, and culturally responsive communication strategies. Future research should evaluate both the effectiveness and implementation of policies and communication strategies at the federal, state, and local levels, including the experiences of recipients of summer meals.

Future research could simulate how and to what extent COVID-19 relief efforts, coupled with equitable living wages, enabled school food service professionals and other low-wage earners to meet the costs of daily living standards and achieve food security [70,71]. Our studies offer some insights into challenges with staff retention, as well as insights into how to address staffing gaps equitably. Many school food service professionals do not make enough money to support themselves and their families and qualify for federal nutrition assistance programs [72]. Increasing resources for school meal staff can have a ripple effect of providing security to families who rely on those staff to prepare and disseminate meals.

### 4.2. Strengths and Limitations

The strengths of this review are in the robustness of the study design and data analysis, which enabled a comprehensive representation of the existing qualitative evidence on emergency school meal programs during the COVID-19 pandemic. This review followed the PRISMA guidelines, ensuring a rigorous and systematic approach to study selection and data extraction processes. To enhance the accuracy of the results, all data extraction was cross-checked by verifying and confirming the extracted data against the original included studies, ensuring that the key themes and information were accurately represented. This approach reduced the likelihood of errors or misinterpretations during the synthesis of the qualitative data.

This scoping review has some limitations. First, while qualitative data allows for a rich exploration of the challenges, experiences, and perspectives of stakeholders involved in the implementation of school meal programs and provides real-time insights during the rapidly evolving COVID-19 pandemic, it is possible that including quantitative studies could help support (or, importantly) contradict our findings. As quantitative studies looking back at this period proliferate, future reviews of the full body of literature are needed. Second, we did not conduct a quality assessment of included studies. The purpose of this review was to map the evidence in a broad and comprehensive manner as opposed to assessing the risk of bias and/or evaluation of the strength of evidence. Many of the included studies were rapidly conducted during the COVID-19 pandemic with the intent to disseminate best practices for meal distribution and to inform ever-evolving policies; as a result, there may have been a methodological trade-off and/or time and/or resource limitations. We aimed for comprehensiveness over quality assessment and thus included both peer-reviewed and grey literature. This approach helped us to include a broad range of evidence. Finally, it is important to note that only three studies in this review offer the perspectives of children, families, or caregivers (all three studies recruited low-income or historically marginalized or minoritized groups). As the recipients and key beneficiaries of school meal programs, children and families are a key voice in informing practical, actionable policy recommendations that better align program operations with their needs. Engaging families, particularly those most at risk for food and nutrition insecurity, in research is aligned with the virtues of reciprocity and community-based practice [31,32,73]. Although these data were understandably difficult to collect during the initial pandemic months, future research should prioritize the important voices of families to advance equity among school meal programs.

## 5. Conclusions

The scoping review provides valuable insights into the operations and utilization of emergency school meal programs during the COVID-19 pandemic. Our findings highlight the immense challenges faced by school food service directors, such as ever-evolving policy changes, competing needs and priorities of families, and overhauled staffing and operational protocols. Findings also highlight the innovative approaches that the directors used to maintain program operations and to provide meals to families who may have previously faced barriers to participation during out-of-school time. Application of the GTE framework revealed that studies of emergency school meal programs reported on equity considerations to varying degrees. Future research should intentionally incorporate health equity frameworks into data collection and analysis, evaluate how emergency school meal programs impact non-food-related social and structural barriers to economic and food security, and assess how policies (such as the recission of the USDA waivers in 2022) affect program operations and implementation. For example, as more states adopt policies to make meals universally free, researchers should monitor policy adoption and implementation to ensure that the meals are more accessible to the hardest-to-reach children. By addressing challenges and implementing equitable solutions, school meal programs have the potential to close the health gaps between those more and less affected by structural and social barriers while improving the health of the population overall.

## Figures and Tables

**Table 1:** Data Extracted from Studies Included in Review (*n* = 21).

Author and Date	Title	Study Aim(s)	Methods	Data Collection Period	Study Location	Study Population
McLoughlin et al, 2020 [8]	Addressing Food Insecurity through a Health Equity Lens: a Case Study of Large Urban School Districts during the COVID-19 Pandemic	To investigate the emergency school meal service strategies in four of the largest school districts and to evaluate the degree to which districts promoted equitable access to emergency nutrition programming in the USA at the beginning of the COVID-19 pandemic.	Content analysis; open and iterative coding; inductive analysis based on GTE framework	March 2020 to May 2020	California, Illinois, New York, Texas	N/A—Document analysis and geospatial analysis of 4 meal site locations
McLoughlin et al, 2020 [35]	Feeding Students During COVID-19-Related School Closures: A Nationwide Assessment of Initial Responses	To assess child nutrition administrative agency responses to meal service provision nationally during COVID-19-related school closures.	Web-based recruitment; systematic open coding; inductive analysis; deductive coding	March 2020 to May 2020	National sample	N/A—Government website data (*n* = 57)
Sharma et al, 2020 [36]	Social determinants of health-related needs during COVID-19 among low-income households with children	To better understand the ongoing needs of families and provide critical services during the pandemic.	Phone-based recruitment; open-ended surveys; thematic analysis; deductive coding	April 2020	Florida, Washington, Texas	Families enrolled in coordinated school-based nutrition program (*n* = 1048)
Chrisman and Alnaim, 2021 [37]	Resources needed for education and meal programs by urban school teachers and staff during the 2019 coronavirus pandemic	To examine teaching needs for teachers and resources needs for meal programs because of COVID-19 for the 2020–2021 school year.	Email-based recruitment; open-ended surveys; content analysis	October 2020	Kansas	School food service directors (*n* = 47), school administrators (*n* = 19), teachers (*n* = 17)
Jablonski et al., 2021 [38]	Emergency Food Provision for Children and Families during the COVID-19 Pandemic: Examples from Five US Cities	Aim 1: To document local responses to the pandemic that support households with K-12-aged children after school closures. Aim 2: To examine the policy and programming consequences of different emergency food interventions to understand their effectiveness in providing emergency food to children and families in need.	Voluntary, purposive sampling; focus groups, interviews, and records/archival review; structural coding method	June 2020 to July 2020	Colorado, Michigan, New York, Ohio, Texas	Emergency food task force members
Jowell et al, 2021 [39]	Mitigating childhood food insecurity during COVID-19: a qualitative study of how school districts in California’s San Joaquin Valley responded to growing needs	To explore best practices and challenges in providing school meals during COVID-19 in an urban–rural region of California.	Phone-, web-, and email-based recruitment, focus groups and interviews, thematic analysis based on social ecological model	June 2020 to August 2020	California	School food service directors (*n* = 12), school superintendents (*n* = 12), partner organizations (*n* = 4), caregivers (*n* = 29)
Kenney et al, 2021 [40]	Feeding Children and Maintaining Food Service Operations During COVID-19: A Mixed Methods Investigation of implementation and Financial Challenges	To estimate the impact of COVID-19 school closures on school food service costs, revenue, and meal service capacity in large urban school districts. Additionally, to assess SFA administrator perspectives on implementing school meal distribution programs during school closures and subsequent reopenings.	Interviews and records/archival review; framework analysis approach; deductive and inductive coding	October 2020 to January 2021	School districts in largest urban US School Food Authorities including the South (*n* = 7), Northeast (*n* = 3), West (*n* = 1), and Midwest (*n* = 1)	School food directors and staff (*n* = 12)
Nikfarjam, 2021 [41]	The Impact of COVID-19 Emergency Flexibilities and Waivers on Child Nutrition Programs in Massachusetts	To analyze the impact of waivers and flexibilities on meal service in schools and food service directors and assess their potential implications for future school nutrition programs.	Interviews; no information on analysis	Unknown	Massachusetts	School food service directors (*n* = 12), directors of community-based organizations (*n* = 5)
Patten et al, 2021 [42]	Disaster Management and School Nutrition: A Qualitative Study of Emergency Feeding During the COVID-19 Pandemic	To evaluate the actions of school nutrition employees during emergency feeding services in the COVID-19 pandemic using the Sendai Framework for Disaster Risk Reduction.	Email recruitment; purposive sampling; interviews; phenomenological analysis	April 2020 to May 2020	All 7 USDA regions	School food service directors (*n* = 34)
Patten et al, 2021 [43]	School Nutrition Professionals’ Employee Safety Experiences During the Onset of the COVID-19 Pandemic	To explore the personal/employee safety experiences and perspectives of school nutrition professionals nationally in the initial weeks of the pandemic.	Web-, and email-based recruitment; open-ended surveys; thematic analysis	March 2020 to April 2020	All states except Hawaii, Alaska, Delaware, and DC	State agency directors (*n* = 13), state agency personnel (*n* = 9), school food service directors (*n* = 214), managers (*n* = 104), supervisors (*n* = 34), staff (*n* = 78)
Bach, 2022 (Center for Science in the Public Interest) [44]	Implementation of the Nationwide Waiver to Allow Specific School Meal Pattern Flexibility for School Year 2021–2022	To examine how the School Meal Pattern Flexibility affected state agency abilities to serve nutritious meals to students during the school year.	Phone- and email-based recruitment; open-ended surveys; no information on analysis	November 2021 to January 2022	National sample	State agency directors (*n* = 23)
Beckstead et al, 2022 [45]	School Nutrition Professionals’ Experiences with Food Safety and Special Diets in School Meals during the Initial COVID-19 Pandemic	To understand the experiences of school nutrition professionals regarding food safety during the initial COVID-19 response.	Web- and email-based recruitment; open-ended surveys; inductive thematic analysis	March 2020 to April 2020	All states except HI, AK, DE, and DC	State agency directors (*n* = 13), state agency personnel (*n* = 9), school food service directors (*n* = 214), managers (*n* = 104), supervisors (*n* = 34), staff (*n* = 78)
Bennett et al, 2022 [46]	Distributing Summer Meals during a Pandemic: Challenges and Innovations	To identify pandemic-related challenges and new practices designed to increase participation in USDA meal programs in Connecticut during the summer of 2021.	Email-based recruitment; semi-structured key informant interviews; phenomenological qualitative approach and immersion crystallization approach	June 2020 to July 2020	Connecticut	School food service directors (*n* = 16)
Braun et al, 2022 [47]	Maintaining School Foodservice Operations in Ohio during COVID-19: “This [Was] Not the Time to Sit Back and Watch”	To characterize COVID-19-related foodservice adaptations among public schools across the state of Ohio.	Email-based recruitment; open-ended surveys; thematic analysis	December 2020	Ohio	School food service directors (*n* = 209)
Bylander et al, 2022 (Food Research and Action Center) [48]	Large School District Report: Operating School Nutrition Programs During the Pandemic	To emphasize the significance of waivers in supporting school nutrition operations and meal access and the need to extend these waivers to ensure all children have access to nutritious school meals through the 2022–2023 school year.	Web- and email-based recruitment; open-ended surveys; no information on analysis	December 2021 to February 2022	31 states (AL; AK; AZ; AR; CA; CT; DE; FL; GA; HI; ID; IL; IN; IA; KS; LA; MD; MA; MI; MN; MO; NE; NV; NJ; NM; NY; NC; OH; OK; OR; PA; RI; SC; TN; TX; UT; VA; WA; WI)	School food service directors (*n* = 62)
Cadenhead et al., 2022 [49]	Qualitative Study of Participation Facilitators and Barriers for Emergency School Meals and Pandemic Electronic Benefits (p-EBT) in an Urban Setting during COVID-19	To better understand emergency school meal participation facilitators and barriers for families in NYC as well as their experiences using P-EBT funds to increase nutrition security during future emergencies.	Web- and email-based recruitment and flyers placed in neighborhoods with high levels of socioeconomic and health challenges; surveys and focus groups; thematic analysis	April and May 2021	New York City	Caregivers (*n* = 126 survey; *n* = 101 focus groups)
Katz et al., 2022 [50]	“Let’s use this mess to our advantage: calls to action to optimize school nutrition programs beyond the pandemic”	To describe multi-level contextual factors that influence program operations to identify factors to address and/or leverage post-pandemic to improve programs.	Email-based recruitment with state agency support; semi-structured key informant interviews; hybrid inductive/deductive phenomenological thematic analysis	May to August 2020	North Carolina	School food service directors (*n* = 23)
Kelley et al, 2022 [51]	Repeated Cross-Sectional Surveys of Registered Dietitian Nutritionists Demonstrate Rapid Practice Changes to Address Food Insecurity During the Coronavirus Disease 2019 Pandemic	To better understand the role of RDNs who were involved in food security efforts during the COVID-19 public health emergency in responding to the crisis, identify challenges they faced, and determine ongoing and future needs.	Email-based recruitment; open-ended surveys; manual review and theme development	Wave 1: April 2020 to May 2020 Wave 2: December 2020 to February 2021	National sample	Community-based registered dietitian nutritionists (Wave 1: *n* = 454, Wave 2: *n* = 331)
Lu et al, 2022 [52]	Serving Summer Meals During the COVID-19 Pandemic: A Case Study of 2 Summer Food Service Program Sponsors in Maryland	To explore and compare the factors to dramatically increase meal distribution during the pandemic at Summer Food Service Program sites (*n* = 2).	Web-based recruitment; interviews; inductive and deductive coding; thematic analysis	September 2020 to February 2021	Maryland	In-depth interviews with (*n* = 4) school food service directors and (*n* = 1) vendor
Payán et al, 2022 [53]	Rural Household Food Insecurity among Latino Immigrants during the COVID-19 Pandemic	To examine the impact of COVID-19 on the household food environments of Latino immigrants in rural communities, especially the role of nutrition assistance resources in mitigating food insecurity during the pandemic.	Convenience sampling; community-engaged approach; interviews; grounded theory; inductive and deductive coding	July 2020 to April 2021	California	Latino immigrants from agricultural communities (*n* = 31)
School Nutrition Association, School Nutrition Foundation, and No Kid Hungry, 2022 [54]	Staying Afloat in a Perfect Storm: The K-12 School Nutrition Segment Contends with Historic Supply Challenges	To collect information on challenges, creative solutions, requested areas for support, and recommended actions regarding the supply chain of K-12 school meal programs.	Web-based recruitment; focus groups; no information on analysis	May 2020	National sample	School food service directors (*n* = 222), distributors (*n* = 10), state agency officials (*n* = 25)

## Data Availability

Full extraction tables and other data from review can be made available upon request.

## Supplementary Materials

The following supporting information can be downloaded at: https://www.mdpi.com/article/10.3390/nu15173738/s1, Supplementary Materials for search strategy are available as a Supplementary File (Supplementary File S1).
