# Supplementary material for: Challenges and Opportunities for Equity in US School Meal Programs: A Scoping Review of Qualitative Literature Regarding the COVID-19 Emergency
Source: Nutrients. 2023 Aug 26;15(17):3738. doi: 10.3390/nu15173738 (PMC10490348; doi:10.3390/nu15173738)
Supplement: Supplementary file 1 [file nutrients-15-03738-s001.zip › nutrients-2539250-SI.pdf]

Supplementary File S1 – Search Strategy

Search strategy for parent review, accepted by PLOS One *(no reference available at this time, 8/25/2023)*

PubMed

Search Terms:

("Supplemental Nutrition Assistance Program"[tiab] OR WIC[tiab] OR "Farmers' Market Nutrition Program" [tiab] OR "Summer Food Service Program" [tiab] OR "Seamless Summer Option" [tiab] OR "Summer meals" [tiab] OR "Summer nutrition program" [tiab] OR "summer feeding program" [tiab] OR "National School Lunch Program" [tiab] OR "School Breakfast Program"[tiab] OR "School meals" [tiab] OR "Special Milk Program" [tiab] OR "Fresh Fruit and Vegetable Program" [tiab] OR "Child and Adult Care Food Program" [tiab] OR CACFP[tiab] OR "Commodity Supplemental Food Program" [tiab] OR "Food Distribution Program on Indian Reservations" [tiab] OR "Farmers to families food box[tiab]" OR "Food Assistance" [tiab] OR TFAP[tiab] OR USDA[tiab] OR "Nutrition Assistance" [tiab] OR "pandemic electronic benefit transfer[tiab]" OR P-EBT[tiab] OR "nutrition assistance" [tiab] OR "food assistance" [tiab] OR "food benefit*" [tiab] OR "food aid" [tiab] OR "food insecurity" [tiab] OR "hunger" [tiab] OR "food policy" [tiab] OR "nutrition policy" [tiab] OR "Food Assistance"[Mesh])

AND

(COVID-19[tiab] OR Coronavirus[tiab] OR SARS-CoV-2[tiab] OR "COVID-19"[Mesh] OR "SARS-CoV-2"[Mesh] OR "Pandemics"[Mesh])

Limits

Published since March 1, 2020; English language

CINHAL, Scopus, and Proquest's Health Management database

Search Terms

("Supplemental Nutrition Assistance Program" OR WIC OR "Farmers' Market Nutrition Program" OR "Summer Food Service Program" OR "Seamless Summer Option" OR "Summer meals" OR "Summer nutrition program" OR "summer feeding program" OR "National School Lunch Program" OR "School Breakfast Program" OR "School meals" OR "Special Milk Program" OR "Fresh Fruit and Vegetable Program" OR "Child and Adult Care Food Program" OR CACFP OR "Commodity Supplemental Food Program" OR "Food Distribution Program on Indian Reservations" OR "Farmers to families food box" OR "Food Assistance" OR TFAP OR USDA OR "Nutrition Assistance" OR "pandemic electronic benefit transfer" OR P-EBT OR "nutrition assistance" OR "food assistance" OR "food benefit*" OR "food aid" OR "food insecurity" OR "hunger" OR "food policy" OR "nutrition policy")

AND

(COVID-19 OR Coronavirus OR SARS-CoV-2 OR "SARS-CoV-2")

CINHAL Limits

Published since March 2020; English language; Source Type – academic journals

Scopus Limits

Published since 2020; English language; Source Type - journal

Proquest's Health Management Database Limits

Published since March 1, 2020; English language; Source Type – scholarly journals
